# Handmade snare technique for grasping and repositioning biliary stents via the endoscopic ultrasound-guided hepaticojejunostomy route

**DOI:** 10.1055/a-2791-4550

**Published:** 2026-02-17

**Authors:** Daisuke Namima, Hideki Kamada, Yoshio Shimizu, Naoki Fujita, Hiroki Yamana, Kiyoyuki Kobayashi, Hideki Kobara

**Affiliations:** 1 12850 Department of Gastroenterology and Neurology, Faculty of Medicine, Kagawa University, Kagawa, Japan; 2 12850 Department of Gastroenterological Surgery, Faculty of Medicine, Kagawa University, Kagawa, Japan

Endoscopic ultrasound-guided hepaticogastrostomy/hepaticojejunostomy (EUS-HGS/HJS) is an established alternative to endoscopic retrograde cholangiopancreatography (ERCP) in patients with surgically altered anatomy 1 2 . When combined with antegrade transpapillary stenting (AGS), dual-route biliary drainage can be secured 3 4 ; however, troubleshooting often requires balloon-assisted endoscopy.

**Fig. 1:**
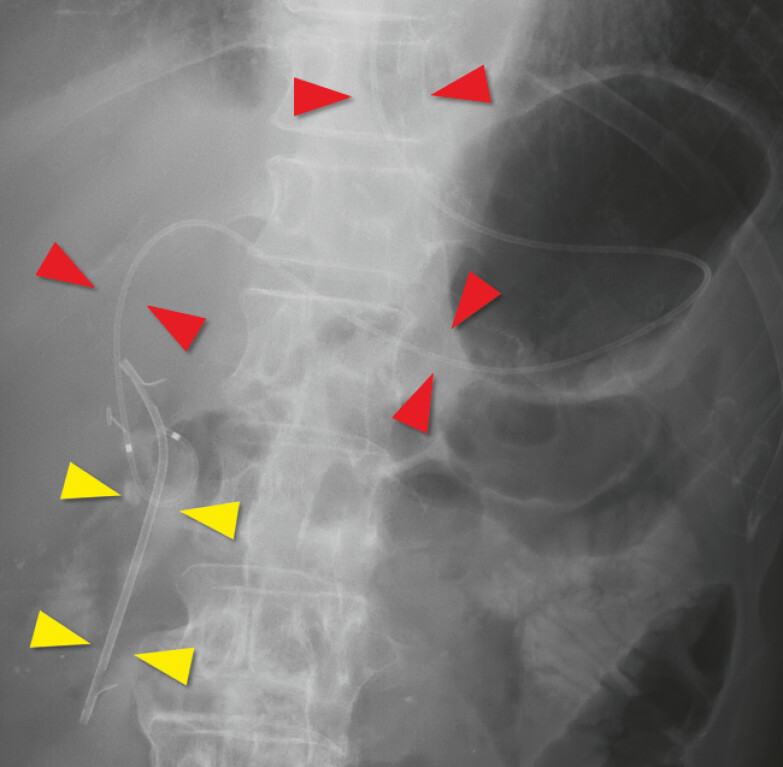
A fluoroscopic image showing an antegrade plastic stent placed across the papilla with its distal end in the duodenum (yellow arrowheads) and an endoscopic nasobiliary drainage tube traversing the endoscopic ultrasound-guided hepaticogastrostomy route (red arrowheads).

A 65-year-old man with surgically altered anatomy and multiple comorbidities presented with septic shock due to choledocholithiasis-related cholangitis. Emergency EUS-HJS was performed to achieve rapid decompression because definitive stone extraction was not feasible at that time. A plastic stent was placed antegradely across the papilla (AGS), and an endoscopic nasobiliary drainage (ENBD) tube was added ( Fig. 1 ).

**Fig. 2:**
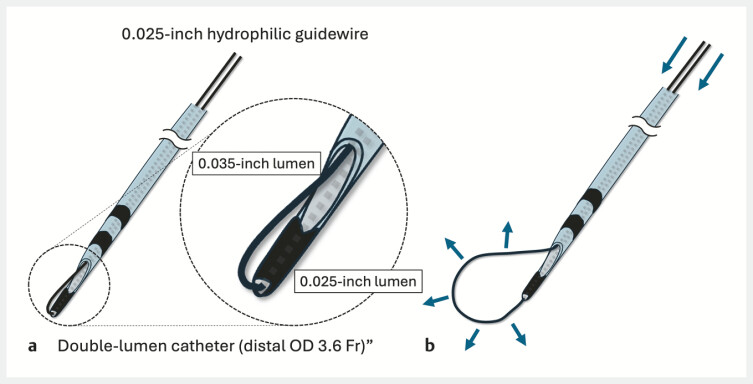
Schema of the handmade snare constructed from an uneven double-lumen catheter. **a** The two ends of a guidewire are inserted through the two lumens of the catheter to form a distal snare loop. The loop is kept closed so that it does not interfere with insertion. **b** Pushing the guidewires from the proximal side advances and opens the snare loop, forming a grasping device.

**Fig. 3:**
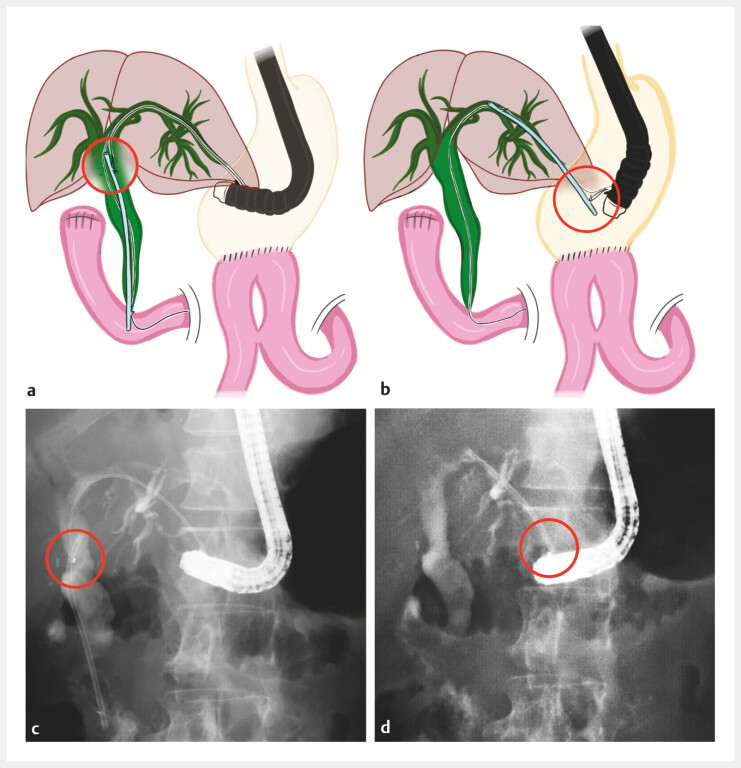
Repositioning a plastic stent via the endoscopic ultrasound-guided hepaticogastrostomy or hepaticojejunostomy (HGS/HJS) route using the handmade snare. In all panels, the grasping portion of the plastic stent is indicated by red circles. **a** Schema showing the distal end of the plastic stent across the papilla being grasped with the handmade snare advanced through the endoscopic ultrasound-guided HGS/HJS route. **b** Schema showing the plastic stent being pulled back and repositioned to the HGS/HJS anastomosis. **c** A fluoroscopic image corresponding to ( **a** ), showing grasping of the plastic stent with the handmade snare. **d** A fluoroscopic image corresponding to ( **b** ), showing the plastic stent relocated to the HGS/HJS anastomosis.

Two weeks later, ENBD cholangiography confirmed stone clearance, and internalization was planned because fistula maturation was uncertain and reintervention without balloon-assisted endoscopy was desirable at the receiving hospital; stent exchange via the HJS route was planned if cholangitis recurred. A standard guidewire was advanced via the ENBD and left in place as a rescue wire in case the stent migrated completely into the bowel; the ENBD tube was removed. A handmade snare was advanced through the HJS tract alongside the rescue wire. As previously reported 5 , the snare was constructed by inserting both ends of a 0.025-inch flexible hydrophilic guidewire commonly used for ERCP in Japan (Radifocus; Terumo, Japan) into the two lumens of a double-lumen catheter (Uneven Double Lumen Cannula; Piolax Medical Devices, Japan; distal outer diameter: 3.6 Fr). Due to the limited wire length, the proximal end of the catheter was cut to externalize both wire ends, creating a soft loop with the wire exiting from the two distal holes ( Fig. 2 ). The loop was used to grasp the stent flap and reposition the stent to the HJS anastomosis; patency was confirmed ( Fig. 3 , Video 1 ).

Handmade snare–assisted repositioning of an antegrade transpapillary plastic stent via the endoscopic ultrasound-guided hepaticojejunostomy (EUS-HJS) route, achieving internalization and preserving biliary access without balloon-assisted enteroscopy.Video 1

This HJS-based technique provides a simple approach for transpapillary stent management without the need for balloon-assisted endoscopy.

Endoscopy_UCTN_Code_CCL_1AF_2AF_3AB Endoscopy_UCTN_Code_TTT_1AR_2AZ
